# Does Physical Interaction with Insight Problems Really Affect the Solution Rate?

**DOI:** 10.3390/jintelligence14050082

**Published:** 2026-05-09

**Authors:** Laura Macchi, Daniele Inglese, Laura Caravona

**Affiliations:** Department of Psychology, University of Milano-Bicocca, 20126 Milano, Italy; d.inglese1@campus.unimib.it (D.I.); laura.caravona@unimib.it (L.C.)

**Keywords:** insight problem solving, physical interaction, misunderstanding, embodied cognition, STEM education

## Abstract

Insight problems are traditionally presented in verbal or visual form; however, some research suggests that physically interacting with the problem materials may facilitate insight problem solving. In contrast, the more recent literature found no performance improvement with manipulable materials, except for spatial problems (e.g., the “Eight Coins problem”). In Experiment 1, to challenge the hypothesis that the facilitating effect of material manipulation is mainly related to spatial insight problems, two groups of participants were presented with another spatial insight problem, the “Pencil problem” (a new version of the traditional “Match problem”), either in paper format or in a physical interactive condition. The results showed no significant differences in success rates based on presentation format. Therefore, manipulating the materials does not appear to facilitate solving spatial insight problems *per se*. In accordance with the misunderstanding theory of insight problem solving, the interacting effect may be limited to situations in which manipulation allows individuals to grasp functional aspects that are crucial for restructuring and reaching the solution. To test this hypothesis, we ran a further study (Experiment 2) in which two groups of participants were presented with a “Matchstick Arithmetic problem” in paper format; one group received the standard representation of the task (I = II + II), while the other, a picture of the arithmetic equation with real matchsticks, whose realistic representation indirectly clarified the misunderstanding that caused the ‘problem forming’ and the impasse. There was a significant increase in solutions in the second condition. In this case, the presence of overlapping matchsticks forming the plus operator helps the participants understand that arithmetic symbols, as well as numbers, can be decomposed, thereby overcoming the misunderstanding that lies in the problem’s critical points.

## 1. Introduction

The study of insight problem solving has its roots in the Gestalt School, when the concept of restructuring was first introduced. This consists of reorganising one or more elements of a problem, which occurs following a state of impasse experienced by the subject attempting to solve the problem and which causes a qualitative change in the representation of the problem itself, with the consequent possibility of arriving at a solution (e.g., Duncker, 1945; Wertheimer, 1959; Öllinger et al., 2008; Gilhooly et al., 2015; and Salvi et al., 2024).

The restructuring process, a change in the representation of the problem, does not address all problems because different types of problems require different approaches. Problem-solving research distinguishes between two types of problems: insight problems, which require a reorganisation of available information, and non-insight or analytical problems, which can be solved through procedural processes (e.g., Schooler et al., 1993; and Gilhooly et al., 2018). In non-insight problems, difficulties can be attributed to the presence of complex calculations or procedures, and according to Simon (1979), the model that best describes the solution-seeking process in these cases is that of the labyrinth. The solution process involves exploring the problem space to find a path to the desired goal, while backtracking whenever a dead-end is reached. In insight problems, on the other hand, the difficulty is associated with one or more critical points, which are the result of a misunderstanding of the participant’s construction of a misleading representation of the problem (Mosconi, 2016). In these cases, reaching a solution requires a change in the representation of the problem, which tends not to happen gradually but rather occurs suddenly.

Several studies have demonstrated significant improvements in performance when an insight problem is presented in an interactive format compared to when participants tackle the same problem in a traditional manner (verbally or visually; e.g., Fioratou & Cowley, 2009; Henok et al., 2020; Vallée-Tourangeau, 2017, 2026; Vallée-Tourangeau et al., 2011, 2016; and Weller et al., 2011). These findings have contributed to the development of the ecological perspective in the field of insight problem solving focused on the interaction between organism and environment (e.g., Steffensen & Vallée-Tourangeau, 2018). This approach argues that objects are not mere carriers of information, nor is the individual a passive processor of information, but rather that a fundamental role is played by the extensive system of relationships that is created between the individual and the objects. According to us, however, it is important to emphasise that in some cases, it may be the information conveyed by the objects that allows the restructuring process of a problem, rather than the interaction that takes place between the individual and the problem material.

In our view, insight problems arise from a misunderstanding (Bagassi & Macchi, 2016) in which the initial representation of the problem leads to an impasse, preventing the solution from being found. The restructuring process takes the form of a reinterpretation of the relationships between the elements and the aim of the problem, contributing to its solution. If we consider misunderstanding to be a fundamental element of the ‘problem forming’ in an insight problem, it follows that presenting a version of the problem stripped of its misleading component should allow a representation of the problem that is functional in reaching the solution. For this reason, we argue that manipulating the material does not help resolve the insight problem *per se* unless it is functional in overcoming the misunderstanding.

Two experiments were conducted to investigate this hypothesis. Experiment 1 challenged previous findings on the facilitating effect of the material on insight problems of a spatial nature (Chuderski et al., 2021). We proposed a spatial insight problem to two groups of participants in paper format or in a physical interaction condition. We expected that there would be no effect from the material manipulation on problem resolution *per se*, even for insight problems of a spatial nature, when the manipulation is not functional for overcoming the misunderstanding. In Experiment 2, we tested the hypothesis that information conveyed by object representation can improve performance, even in the absence of the material itself, by presenting a realistic picture of the problem elements that indirectly clarifies the misunderstanding that caused the impasse. Our prediction was that this information would be sufficient to overcome the misunderstanding and solve the insight problem.

### 1.1. The Ecological Perspective on Insight Problem Solving

Insight problems are traditionally presented in verbal or visual form, without the option to physically interact with the problem material. However, several studies have shown significant improvements in performance when a problem is presented in an interactive format (e.g., the “Cheap Necklace Problem”, Fioratou & Cowley, 2009) compared to when participants work on the same problem in a static format. These results have contributed to the development of the ecological perspective focused on the interaction between organism and environment (e.g., Steffensen & Vallée-Tourangeau, 2018).

One of the first studies on the role played by material manipulation during the process of solving an insight problem was conducted by Fioratou and Cowley (2009). The two authors show that in the “Cheap Necklace problem”^1^ participants exhibit significantly better performance when they have the possibility to manipulate the physical rings that form the chains than when they are in a “paper-and-pencil” condition (30% vs. 3% of solutions). This improvement in performance is not attributed to the presence of the objects themselves (or to an externalised representation of the problem), but rather to the possibilities of actions and perceptions that the objects themselves offer. Indeed, when the authors propose a new version of the problem to the participants in which the two external rings of a chain are presented as open, with the aim of making the possibility of “breaking the entire chain” more salient, there is a further improvement in performance. Therefore, if differences in the resolution percentages are observed between the condition with open rings and that with closed rings (66% vs. 30%), regardless of the presence of the objects, then the authors report the following:

[…] solutions depend on neither artefacts themselves nor what we see. While environmental factors contribute to solutions, external representations are less important than monitoring the possible effects of actions. Where events in a problem space set off insights, solutions arise from perceiving possibilities and noting the effects of current actions. We depend on experience.

(Fioratou & Cowley, 2009, p. 563)

Other evidence supporting the idea that the possibility of physically interacting with the elements of an insight problem can facilitate the achievement of the solution comes from the work conducted by the research group of Vallée-Tourangeau. These studies, in particular, report significantly higher percentages of resolution in an interactive condition, compared to a static one, as is seen in some “Matchstick Arithmetic problems” (Weller et al., 2011), in the “Water Jars problem” (Vallée-Tourangeau et al., 2011), in the “17 Animals in Pens problem” (Vallée-Tourangeau et al., 2016) and in the “Triangle of Coins problem” (Vallée-Tourangeau, 2017). According to Vallée-Tourangeau (2014), in fact, “artefacts that constitute the physical model of the problem guide and constrain the manner with which problem-solving behaviour is enacted” (p. 39). The author points out that objects are not mere carriers of information, nor the individual a passive processor of information, but, rather, a fundamental role is played by the widespread system of relationships that is created between the individual and the objects. In our opinion, it is important, however, to point out that in some cases it may be the information conveyed by objects that favours the achievement of the solution of a problem, rather than the interaction that takes place between the individual and the material of the problem. For example, with regard to the “Matchstick Arithmetic problems”^2^, the possibility of decomposing the “+” operator, and not just the numbers, could be communicated more effectively when the operator is represented with overlapping sticks (see Experiment 2) than when the equation is presented in a stylised form (I = II + II). This information, alone and independent of the subsequent manipulation of objects, could, therefore, be sufficient to improve the performance of the participants.

In any case, among the advantages related to the presentation of a physical model of the problem, Vallée-Tourangeau (2014, 2017, 2026) also reports the consequent reduction in the workload of working memory, since participants can enact real actions on the elements of the problem, instead of mentally simulating them. For these reasons, the author concludes that, just as in everyday life, people face problems by making changes through their actions on the world; even in the laboratory, it is appropriate to present problems in more ecological formats, which allow the individual to take action:

[…] problem solving in the world proceeds on the basis of changes in the world: People and researchers alike act on the world, manipulate artefacts and models and rearrange physical features of a problem. This is problem solving. The evidence of successful problem solving can be found in changes in the world. Whether a mental representation was restructured or not is secondary to the behavioural and physical evidence: How interactivity exploits and modifies external resources should first be documented.

(Vallée-Tourangeau, 2014, pp. 40–41)

The results highlighted by these studies seem to be in line with theories of embodied cognition^3^, a perspective that believes that “the body or body’s interactions with the environment constitute or contribute to cognition” (Shapiro & Spaulding, 2025). Engel et al. (2013) report a series of experiments conducted by various researchers in the field of neuroscience and cognitive psychology, which seem to support the idea of a strong relationship between cognition and bodily actions exercised by the individual on the environment. The studies illustrated by the authors suggest that knowledge of objects is not limited to the presence of descriptive representations of them, but also includes sensorimotor knowledge relating to the possibilities for action and use. Consequently, the understanding of cognition cannot be based on the analysis of mental representations or models of the world alone, but the fundamental role played by bodily interactions with the environment in the process of knowledge construction and the development of cognitive processes should be considered.

Based on this theoretical perspective, Thomas and Lleras (2009) tested the role played by body movements in insight problem solving. The participants in the experiment were given the “Two-String problem”^4^. During the attempts to find the solution, the researchers proposed short breaks to the participants: a first experimental group was asked to perform arm swinging movements, linked to the solution of the problem, while a second control group was asked to perform stretching movements, which had no connection with the solution of the problem. The results show significantly better performance in the experimental group than in the control group (85% vs. 62% of solutions): in other words, the implicit experience of a movement linked to the solution would seem to be functional in the achievement of such a solution. Therefore, consistent with the theories of embodied cognition, the authors conclude that an individual’s bodily actions can also influence thought processes during the resolution of an insight problem. However, it should also be noted that, in an a posteriori control condition, in which Thomas and Lleras ask participants to remain still and hold their arms at their sides during breaks, the problem resolution rate stands at 72%. Although for the authors, this percentage may not be directly comparable with that of the experimental group that performed the oscillatory movements, it is evident that, even in the absence of movements that recall the solution, a high number of the participants were able to solve the problem.

Despite the popularity and the attention that embodied cognition theories have received in recent decades of research, some authors have highlighted critical issues in this perspective. Goldinger et al. (2016) analysed a series of cognitive phenomena, such as the effect of word frequency or categorisation, with the aim of evaluating whether the results of some studies of classical cognitive psychology could be better explained from the perspective of embodied cognition: for most of the phenomena analysed, no advantages seem to emerge. The authors believe, in fact, that embodied cognition is often roughly defined and that it is not able to offer new insights for scientific reflection or better explanations than those already provided by classical cognitive psychology. Furthermore, in their overview, Loginov and Spiridonov (2023) have pointed out the insufficient theoretical exploration of the psychological mechanisms underlying the effects discovered in several studies on “embodied problem solving” and the non-comparability of these studies, which are based on different problems and extremely heterogeneous paradigms.

Similarly, despite the multiple pieces of evidence listed above in support of the idea that physical interaction with objects favours the resolution of insight problems, it is also crucial to highlight results in the literature that seem to counter this idea. A study by Vallée-Tourangeau et al. (2020) already shows that the possibility of manipulating the material of a problem does not always lead to significant differences in individual performance. Two experiments were conducted in which the participants were presented with the “Triangle of Coins problem”^5^, in a condition of high or low interactivity: in the high interactivity condition, participants could move coins on a screen, while in the low interactivity condition, they could only look at them without moving them. In the first experiment, as typically observed in the literature, the participants had no limit on the number of solution proposals they could enunciate to the experimenter, while in the second, the participants were bound to be able to propose only one solution. The results show that, at the end of the ten minutes available, when the participants could give only one solution, the performance of the high interactivity group was significantly better than that of the low interactivity group (70% vs. 46% of correct solutions). When, on the other hand, they had no limit in the number of answers (which is the standard request for an experiment on problem solving), despite the fact that in the highly interactive condition, on average, there are a lower number of proposed solutions and a lower latency in the solution times, no significant differences emerged in the resolution of the problem compared to the condition of low interactivity (68% vs. 65%).

In line with these findings, Spiridonov et al. (2026), with the “Matchstick Arithmetic problems”, do not find any difference in solution rates between the interactive conditions and the static condition. They even observe, in the interactive conditions, a negative impact of the number of manipulations on the problem resolution: the participants’ movements do not affect chunk decomposition, but they do significantly hinder the overcoming of high-level constraints.

**Figure 1:**
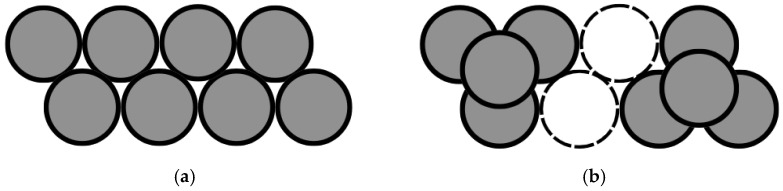
(**a**) The “Eight Coins problem” and (**b**) the “Eight Coins problem” solution.

Chuderski et al. (2021) show even more clearly how material manipulation is not always an advantage for solving an insight problem. First, they attribute the results obtained in the research supporting the ecological perspective on insight problem solving to the limited size of the samples adopted and to the poor performance of the control groups compared with those emerging in other studies on the same problems. For example, the control group in the study by Fioratou and Cowley (2009) solves the “Cheap Necklace problem” in 3% of cases, while in the study by Chu et al. (2007), participants solve the same problem in 43% of cases. Similarly, 22% of solutions from the control group in the “Triangle of Coins problem” (Vallée-Tourangeau, 2017) also appear very different from the 85% found by Nęcka et al. (2016) and the 43% reported by Chuderski and Jastrzębski (2018). Subsequently, Chuderski et al. (2021) conducted a study, in which they administered nine insight problems^6^ to a large sample of participants of varying difficulty in a “paper-and-pencil” condition or in an interactive one. The results show no significant difference in problem resolution rates between the two conditions, with the exception of the “Eight Coins problem” (Figure 1a,b), where there is a significant advantage in favour of the interactive condition. This problem requires participants to move two out of eight coins so that each coin touches exactly three other coins; the coins will need to be separated into two groups.

The solution requires moving one coin on top of three others twice (Figure 1b). The authors hypothesise that, since it is a spatial insight problem, whose solution requires moving from a two-dimensional to a three-dimensional plane, the possibility of physically manipulating coins has promoted a three-dimensional exploration of space and, consequently, the achievement of the solution, while the paper format has constrained a two-dimensional reasoning.

### 1.2. Misunderstanding Theory

Important questions regarding the nature of the role that manipulating material in problem solving arise from the conflicting results present in the literature on interactive insight problem solving mentioned previously. One question in particular is of significant interest: can physical interaction, *per se*, with the objects involved in the problem really be crucial for the process of restructuring the problem? This question takes on even greater significance when considered within the theoretical framework advanced by Macchi and Bagassi (2012, 2015), which identifies misunderstanding as a fundamental element of insight problems. The authors suggest considering “insight problems as paradigmatic examples of misunderstanding, in that they arise from a *qui pro quo*, a glitch in communications.” (Bagassi & Macchi, 2016, p. 60). When the experimenter proposes a problem to the participant, the initial interpretation of the message presented, which derives from an equivocal understanding of one or more elements, does not allow the solution to be found and leads to a state of impasse. In order to get out of this stalemate, the participant must understand that the initial interpretation is not functional for the purpose of the problem and seek a more appropriate one. The restructuring process, in this sense, is configured as a process of reinterpreting the relationships between the elements and objectives of the problem, which contributes to its solution. Reinterpretation does not take place on the basis of abstract reasoning or extremely exhaustive research, but rather is guided by that information, provided by the problem and the context, that is most relevant to the achievement of the solution. During this process, in particular, the intervention of a highly adaptive heuristic of the human cognitive system, i.e., the interpretative function, is fundamental, which allows for maximising the information potential of the available data (Macchi, 2026). This function, in known situations, allows the recognition of family relationships, while, in situations in which habitual relationships are inadequate, it allows and guides the exploration of new meanings and new connections. Interpretive heuristics can come into play in any process related to thought, language and perception: for example, they intervene both during the restructuring of an insight problem and during the reinterpretation of a misunderstood word in a conversation. Precisely because of the use of this heuristic, it is possible to compare the process of solving an insight problem with that which occurs when trying to understand the meaning of a word or an expression following misunderstandings in communication. If we consider misunderstanding as a fundamental element of the ‘*problem forming*’ of an insight problem, then the administration of a version of the problem purified of its misleading component should favour the construction of a representation of the question that is functional in the achievement of the solution. Indeed, Macchi and Bagassi report a significant increase in the number of solutions obtained by participants when they propose some reformulated versions of classic insight problems, such as the “Square and Parallelogram problem”, the “Pigs in a Pen problem”, the “Bat and Ball problem” and the “Study Window problem”, compared to when they provide the original text of the problem.

Consider, for instance, the “Bat and Ball problem”:

*A bat and a ball cost $1.10 in total. The bat costs $1.00 more than the ball. How much does the ball cost?___cents*

The answer which immediately comes to mind is 10 cents, which is incorrect, as, in this case, the difference between $1.00 and 10 cents is only 90 cents, not $1.00 as the problem stipulates. The correct response is 5 cents.

If the rhetorical structure of the text is analysed, the question as formulated concerns only the ball, implying that the cost of the bat is already known. The question gives the key to the interpretation of what has been said in each problem and, generally speaking, in every discourse. Given data, therefore, is interpreted in light of the question. Hence, “The bat costs $1.00 more than” becomes “The bat costs $1.00”, by leaving out ‘‘more than’’. Consequently, we reformulated the text in order to eliminate this misleading inference.

Experimental version:

*A bat and a ball cost $1.10 in total. The bat costs $1.00 more than the ball. Find the cost of the bat and of the ball.*

The difference in the percentages of correct solutions was significant: when participants were asked to find the cost of both objects, instead of limiting the question only to that of the ball, a considerable increase in the number of solvers was observed (90% vs. 10% of solutions). The simple reformulation of the question, which expresses the real aim of the task (to find the cost of both items), removes the misunderstanding of considering the cost of the bat as already known (“$1”), by leaving out part of the phrase (“more than”).

Therefore, the reformulation of the text of an insight problem, when it allows an elimination of the aspects that convey a misleading message and is more relevant to the requested purpose, favours the overcoming of the critical points of the problem. In light of the conflicting results on the effectiveness of the ecological perspective in the field of insight problem solving, it is also interesting to investigate whether and when the presence of objects and their manipulation can help overcome the misunderstanding underlying the formation of the problem.

To further investigate the influence of object manipulation on the solving process of an insight problem, two experiments were conducted. Experiment 1 challenged, in particular, the hypothesis of Chuderski et al. (2021), relating to the idea that the facilitating effect of material manipulation mainly concerns insight problems of a spatial nature, proposing to two groups of participants the “Pencil problem” (a variant of the traditional “Match problem” by Kanizsa, 1978), in paper format or in a condition of physical interaction with the material. We expected to see no effect from the material manipulation on the problem resolution, even with insight problems of a spatial nature, when the manipulation is not functional to overcoming the misunderstanding. Experiment 2, on the other hand, tested the hypothesis that the information conveyed by object representations can produce improvements in performance, even in the absence of the objects and, of course, their manipulation, presenting two groups of participants with a “Matchstick Arithmetic problem” only in a paper format: one group received the standard representation of the task (I = II + II), while the other group received a picture of the arithmetic equation with real matchsticks, which indirectly clarified the misunderstanding that caused the impasse. In this second version, the presence of overlapping matchsticks forming the plus operator helps participants understand that arithmetic symbols, as well as the numbers, can be decomposed, thereby overcoming the misunderstanding that lies in the problem’s critical points. According to our hypothesis, this would allow the misunderstanding to be overcome.

## 2. Experiment 1. The Pencil Problem: Does Interactivity Improve Performance in Spatial Insight Problems?

In Experiment 1, starting with the results from Chuderski et al. (2021), which report an improvement in performance associated with the possibility of physically interacting with the material only in the case of a spatial insight problem, we decided to propose to the participants a problem of this nature, namely the “Match problem” (Kanizsa, 1978), which we renamed the “Pencil problem”, as it has pencils as its object instead of matches. To the control group, the problem was presented in paper format, while, for the experimental group, the text was accompanied by material to be manipulated.

The initial research hypothesis predicted that manipulation would not facilitate the resolution of the problem. This hypothesis was developed taking into account the contradictions in the literature regarding the effectiveness of presenting insight problems in interactive formats and the specific difficulty of the problem used in the study. On the one hand, the series of studies accounts for the idea that physically interacting with the objects of the problem could facilitate the achievement of the solution, encouraging a three-dimensional exploration of space. However, taking the theoretical framework of the studies of Macchi and Bagassi (2015) on the misunderstanding and reformulation of the text, the mere presence of material to be manipulated may not be sufficient to overcome the critical points of the problem.

The original instruction of the “Match problem” (Kanizsa, 1978) was the following:

*With six matches (without bending or breaking them), form four equilateral triangles.*

**Figure 2:**
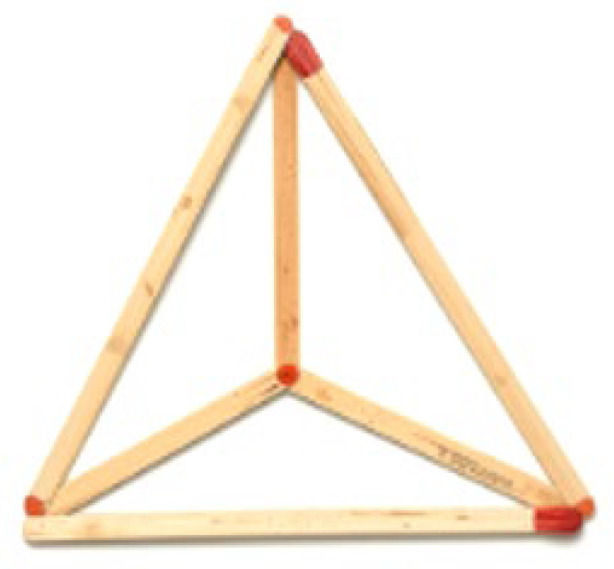
The “Match problem” solution.

By informing the participant of the impossibility of breaking or breaking matches, it is implicitly communicated that the length of each side corresponds to the entire size of the match. It is a spatial insight problem as it cannot be solved through the implementation of known procedures or rules, but rather a qualitative change in its representation is necessary, capable of allowing the overcoming of the constraint that leads to limiting the search for the solution within a two-dimensional space (originating from the two-dimensional nature of the figures subject to the problem). To solve the problem, in fact, it is necessary to explore three-dimensionality and build a tetrahedron (see Figure 2). The percentages of the problem resolution from the samples of university students, in the absence of suggestions from the experimenter, range between 19% and 29% (Reid, 1951).

The resolution of the “Match problem” requires a higher degree of space exploration than the “Eight Coins problem” used in the study by Chuderski et al. (2021). If, in fact, the “Eight Coins problem” is solved by overlapping the coins (where even random manipulation directly provides a hint for the solution), in the “Match problem”, on the other hand, a simple overlapping of the material is not sufficient to achieve the goal presented in the instructions and restructuring.

### 2.1. Methods

#### 2.1.1. Materials and Procedure

**Figure 3:**
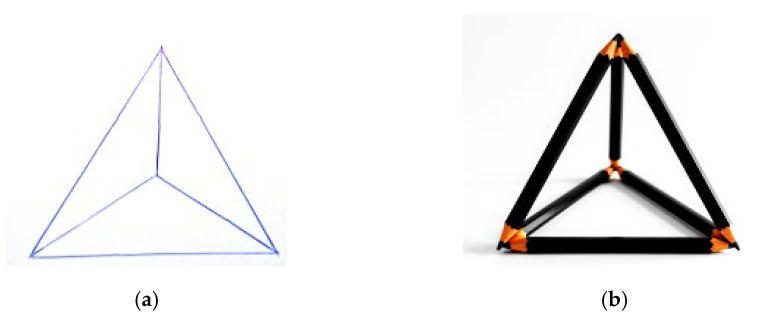
(**a**) The “Pencil problem” solution in the standard condition and (**b**) the “Pencil problem” solution in the interactive condition.

In Experiment 1, matches were replaced with pencils, hence the new naming of the problem as the “Pencil problem”. The problem was presented in two different conditions: a control condition (*n* = 44), in which the problem was presented in paper format, and an experimental condition (*n* = 43), in which, the text of the problem, was accompanied by material to be manipulated: six pencils of equal length (16 centimetres) with which it was possible to physically interact during the search for the solution (see Figure 3).

The instruction of the problem presented to the participants was as follows:

*With six equal pencils (without bending or breaking them), form four equilateral triangles.*

Each participant, tested individually, was asked to read the “Pencil problem” and try to solve it for up to 15 min. Specifically, the problem text was given to the participant with a sheet of paper (A4 size) and a pen (to draw the answer on the sheet) in the control condition, or with six pencils (to build a physical model of the solution) in the experimental one. In the experimental condition, there were no constraints on the use of the pencils, except those explained in the text problem (*bending or breaking the pencils*).

During the experiment, the assignment remained available to the participants, so that it could be read again, and an experimenter was present who observed the behaviour of the participants and recorded the time.

#### 2.1.2. Participants

The study involved a sample of 90 students from the University of Milano-Bicocca, from which 3 participants attending a master’s degree course in psychology were excluded, as the topics of interest for the study were covered. The final sample obtained includes 87 participants aged between 19 and 27 years (*M* = 21.44 years, *SD* = 1.91, range 19–27 years; 39 males and 48 females). Particular attention was paid to including both students from STEM^7^ degree courses (*n* = 45) and students from other degree courses (*n* = 42) in the sample. Participants were assigned to the two conditions in order to ensure a balanced number of STEM and non-STEM students in each group. The recruitment process of participants took place both in the buildings of the university most frequented by the STEM students and in the buildings where classes are mainly attended by students of non-STEM courses. The choice to consider this variable was motivated by the fact that recent studies have shown that STEM education can foster the development of problem-solving skills (Giang et al., 2024) and by the fact that the nature of the solution to the match problem, which consists of the construction of a tetrahedron, is geometric. For these reasons, in fact, it was hypothesised that, regardless of belonging to the control or experimental group, the presence of a STEM-type competence could have favoured better performance.

### 2.2. Results

The “Pencil problem” was solved by 27.3% of the participants (12/44) in the “paper” control condition, and by 32.6% (14/43) in the experimental condition. There were no significant differences between the solution percentages in the two conditions (*χ*^2^ [1, *N* = 87] = 0.290, *p* = .590, and *V* = 0.058). In other words, compared to the “paper” format, the opportunity of manipulating the material involved did not facilitate the process of solving the problem. The lack of effect of this condition on the probability of finding the solution also emerges when the two groups of participants, STEM (*χ*^2^ [1, *N* = 45] = 0.192, *p* = .661, and *V* = 0.065) and non-STEM (*χ*^2^*_LR_* [1, *N* = 42] = 0.228, *p* = .633, and *V* = 0.074), are considered individually.

**Figure 4:**
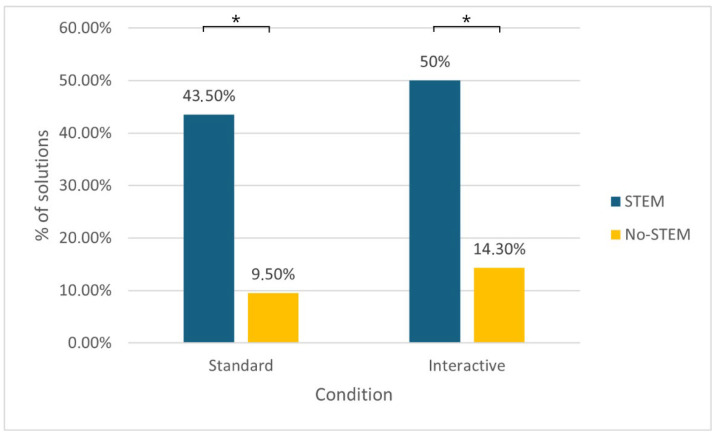
A comparison between the STEM vs. non-STEM percentage of problem solutions in the standard vs. interactive condition.

A greater number of the participants attending the STEM courses solved the problem compared to those attending other courses. In fact, 46.7% (21/45) of the solutions were reached by STEM participants, and 11.9% (5/42) by the non-STEM participants. In this case, the chi-squared test, *χ*^2^ (1, *N* = 87) = 12.528, *p* = < .001, and *V* = 0.379, indicates that the percentage of STEM participants who solved the problem is significantly higher than that of the non-STEM participants. Therefore, it would seem that an education based on STEM studies would facilitate the solution of the “Pencil problem”. The difference in the performance of the two groups is still present when the control condition (*χ*^2^ [1, *N* = 44] = 6.381, *p* = .012, and *V* = 0.381) and the experimental condition (*χ*^2^[1, *N* = 43] = 6.241, *p* = .012, and *V* = 0.381) are examined separately. Figure 4 shows the distribution of the STEM and non-STEM participants who solved the problem in the two conditions, while Table 1 summarises the performance of the entire sample, broken down into the condition (standard or interactive) and the degree programme followed.

The time taken by the participants to construct the tetrahedron was then analysed. On average, in those cases in which the solution was reached, the participants who solved the problem worked on it for 491 s. Taking the two conditions separately, the average time decreases to 455 s for the control group and increases to 523 s for the experimental group. Although the average solution times do not differ significantly between the two conditions (*t* [24] = −0.596, *p* = .556, 95% CI [−304.09, 167.73], and *d* = −0.235)—the assumption of homogeneity of variances was assessed using Levene’s test and was not violated, *F*(1, 24) = 0.025, *p* = .876—the tendency to take more time to solve the problem in the experimental condition is worth noting. Therefore, not only does manipulating the objects involved in the problem not help to solve it more rapidly compared to the “paper” format, but also there was an unexpected tendency to take more time (even though not significantly more) to reach the solution.

**Figure 5:**
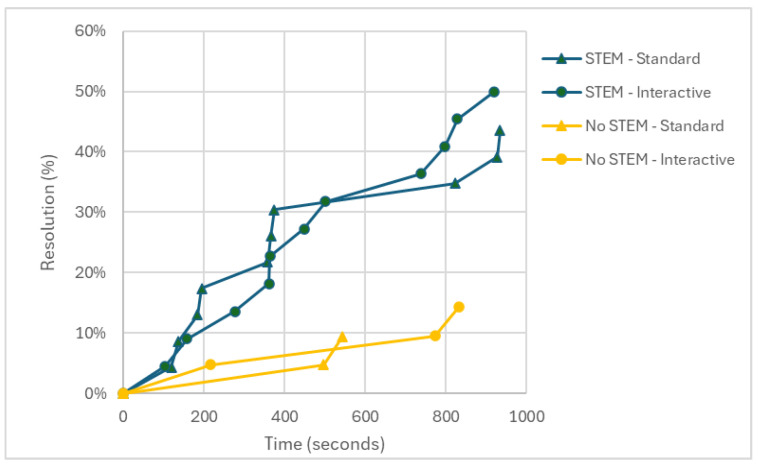
The probability of solving the problem over time for STEM and non-STEM participants in the two conditions.

Finally, a Cox proportional hazards regression model (Cox & Oakes, 1984) was used to examine the influence of *condition* (standard vs. interactive) and *degree programme* (non-STEM vs. STEM) on the probability of reaching the solution within the time limit set by the study. The Cox model makes it possible to estimate the probability that a given event will occur within a specified time frame, while taking into account a set of factors that may influence this probability. In addition, the model also accounts for right-censored data, that is, cases in which the event of interest did not occur during the observation period; accordingly, both solvers and non-solvers were included in the analyses. The results showed no significant differences between the two conditions with respect to the probability of reaching a solution within the time limit (*B* = −0.035, *SE* = .399, *p* = .930, *HR* = 0.966, and 95% CI [0.442, 2.111]). With regard to the degree programme, however, non-STEM participants exhibited a significantly lower probability of solving the problem within the allotted time (*B* = −1.382, *SE* = 0.503, *p* = .006, *HR* = .251, and 95% CI [0.094, 0.673]). Figure 5 illustrates the probability of solving the problem over time for STEM and non-STEM participants in the two conditions.

### 2.3. Discussion

The statistical analyses carried out on the results did not show any significant difference between the performance of the control group and that of the experimental group. Working on an interactive format of the problem, which offered the possibility of manipulating the pencils, did not facilitate reaching the solution. This is true both for the number of participants who solved the problem and for the time taken by the participants to reach the correct solution. In fact, the only variable that had a significant impact on the performance of the participants was the degree programme followed (STEM vs. non-STEM).

The absence of a facilitating effect of physical interaction with the pencils observed in the results of this experiment does not support Chuderski et al.’s (2021) hypothesis, according to which manipulating the objects involved in the experiment should facilitate the solution of spatial insight problems. More specifically, the interactive format did not prove more effective than the paper format in communicating the need to sufficiently explore the problem space in order to construct a three-dimensional solid (the tetrahedron). In line with the initial hypothesis of the present study—developed within the theoretical framework proposed by Macchi and Bagassi—it can therefore be argued that the availability of a physical model did not help participants to better grasp the critical aspect of the problem, that is, the point enabling them to overcome the initial misunderstanding and reinterpret the problem in light of the solution.

The evidence supporting the better performance of the participants following STEM courses that emerged from the data analyses, on the other hand, is in line with studies that suggest the efficacy of a STEM education for enhancing problem-solving competencies (Giang et al., 2024). This might be attributable to the concepts of geometry present in the “Pencil problem”; indeed, STEM participants may have a greater command of and be more familiar with certain elements present in the problem and consequently have more confidence in this area, which would facilitate the task of interpreting the text and executing a more complete and informed analysis. In fact, the geometric nature of the problem may have allowed the STEM participants to identify quickly the presence of a misunderstanding that has to be overcome in order to reach the solution; this would prompt them to initiate a restructuring process immediately and with greater awareness. Almost all the STEM participants realised in the first few minutes that every pencil had to be used contemporaneously as a side of two triangles because otherwise there would not be enough pencils. The verbal reports often contain a reference to this. Below are two examples:

Participant 36: *If I have six pencils and I have to create 12 sides, it means that every pencil has to correspond to 2 sides.*

Participant 52: *Every pencil has to be a side of two triangles*.

In any event, it is worth noting that even among the STEM participants, there was no improvement in performance in the experimental condition, that is, when the participants were provided with pencils to manipulate. In conclusion, even a competence that derives from a STEM background, associated with a greater ability in problem solving, did not enable the participants to benefit from the possibility of manipulating the material involved in the problem. In the “Pencil problem”, compared to the paper format, the physical objects did not convey a representation that was more functional for reaching the solution, regardless of the degree programme the participants were following.

## 3. Experiment 2. The Matchstick Arithmetic Problem: Overcoming Misunderstanding Without Interaction

**Figure 6:**
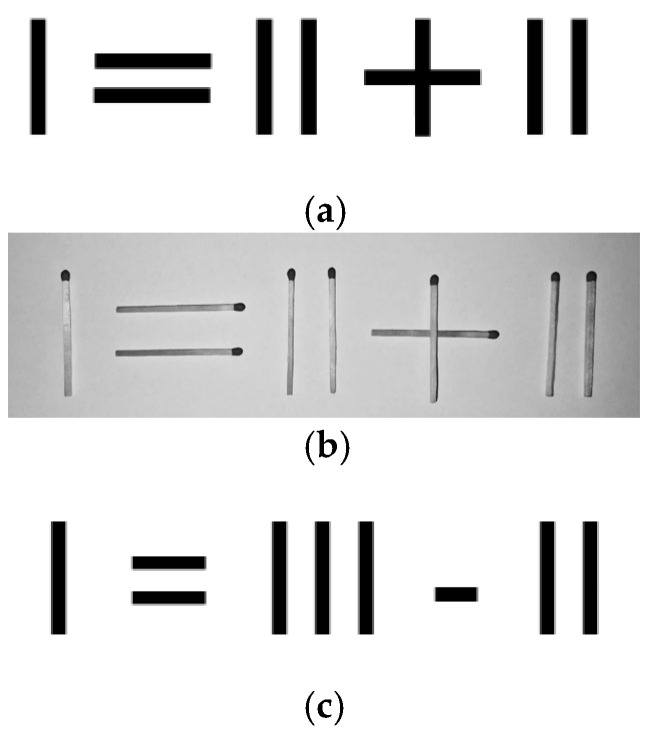
(**a**) The “Matchstick Arithmetic problem” in the standard condition; (**b**) the “Matchstick Arithmetic problem” in the picture condition; and (**c**) the “Matchstick Arithmetic problem” solution.

Following the results of the “Pencil problem” (Experiment 1) regarding the absence of a facilitating effect deriving from the manipulation of the physical objects, we conducted a second study with the aim of further problematising the role of physical interaction with objects in insight problem solving. In this second experiment, Experiment 2, we decided to test the hypothesis that, in certain cases, the misunderstanding could be overcome by showing the participants a photograph of the actual objects involved in a problem, even if they are not physically present and therefore cannot be manipulated; if the information derived from such photographs could be useful for overcoming the misunderstanding, performance on reaching the solution to the problem could improve. We therefore submitted a “Matchstick Arithmetic problem” in paper format to our participants, who were divided into a control group and an experimental group. The control group was given the standard version of the problem—an arithmetical operation constructed with stylised matchsticks—while the experimental group was given a photograph of the same operation, constructed with real matchsticks (Figure 6). As can be seen in Figure 6, the photograph clearly shows that the plus operator has been constructed by placing one match on top of another, implicitly communicating that its components can be moved to solve the problem.

The “Matchstick Arithmetic problems” (Knoblich et al., 1999) consist of false arithmetical operations written in Roman numerals that the participant is asked to correct. Both the numbers and the operators (arithmetical signs) are represented with stylised matchsticks. The participant is allowed to move only one matchstick from one position to another within the operation to obtain the correct result. These problems are bound by self-imposed constrains, which are illustrated in Table 2 below.

### 3.1. Methods

#### 3.1.1. Materials and Procedure

Each participant was tested individually and asked to solve a “Matchstick Arithmetic problem” within a maximum time of three minutes. The problem was presented on paper with the following request:

*You will be presented with a problem with matchsticks. The problem is made up of three Roman numerals and two arithmetic signs. You can move only one matchstick, so that the problem is arithmetically correct. No matchstick can be discarded.*

The control group (*n* = 30) received the standard version, in which the matchsticks were represented in stylised form (Figure 6a), whereas the experimental group (*n* = 29) was presented with a photograph of the same arithmetical operation constructed with real matchsticks (Figure 6b). The two versions were of the same size. The selected problem required relaxation of the operator constraint: to solve the problem, participants had to decompose the plus operator (Figure 6c). This choice was motivated by the hypothesis that the possibility of decomposing the plus operator—rather than focusing exclusively on the numerical elements—would be conveyed more effectively when the operator was represented by a matchstick laid across another, as opposed to the stylised depiction. Crucially, this visual information alone, independent from any physical manipulation of the matchsticks, might be sufficient in overcoming the misconception that the operator cannot be modified, thereby improving participants’ performance.

#### 3.1.2. Participants

The initial sample consisted of 66 students from the University of Milano-Bicocca. Six participants were excluded because they were already familiar with “Matchstick Arithmetic problems”. In addition, the data analysis excluded one participant enrolled in a STEM degree programme (statistics), as all remaining participants were enrolled in non-STEM degree programmes. This participant was excluded in order to ensure sample homogeneity and, in light of the results of Study 1, to avoid the potential influence of STEM-related training on task performance, regardless of assignment to the control or experimental group.

The final sample, therefore, comprised 59 participants aged between 19 and 25 years (*M* = 22.1, SD = 1.17; 20 males, 39 females), all enrolled in non-STEM degree programmes.

### 3.2. Results

**Figure 7:**
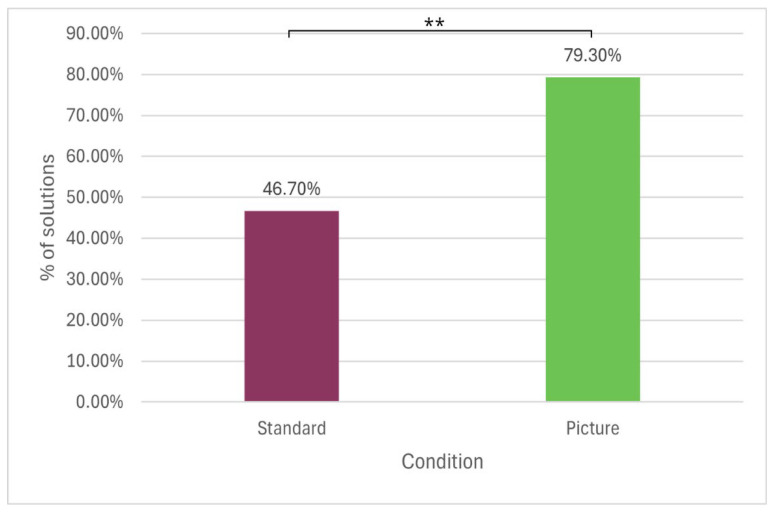
The percentages of the solutions in the standard and picture conditions.

The “Matchstick Arithmetic problem” was solved by 46.7% (14/30) of participants in the control group and by 79.3% (23/29) of those in the experimental group. A chi-squared test, *χ*^2^(1, *N* = 59) = 6.720, *p* = .010, and *V* = 0.337, revealed a significant difference in solution rates between the two conditions. Compared with the stylised version, the presentation of the problem as a photograph of real matchsticks was associated with a higher proportion of correct solutions. Figure 7 illustrates the distribution of solvers across the two conditions.

An additional noteworthy finding emerges from the examination of the responses provided by non-solvers. In the experimental group, the six participants who failed to solve the problem did not produce any alternative solution when the three-minute time limit expired. In the control group, by contrast, nine participants did not reach the correct solution, while seven of them proposed an alternative response (I = III − II). Among these latter participants, six moved a matchstick to modify the “=” sign, thereby producing “≠”. This response was considered wrong, as it was too generic and applicable to any arithmetically correct mathematical operation. However, it is worth noting that this type of response was observed exclusively in the control group and may be attributable to the fact that the standard version of the problem (Figure 6a), more than the experimental version (Figure 6b), appears to suggest the need to identify a formal, mathematically oriented solution. None of the participants in the experimental group came up with this rather uncommon mathematical symbol (≠) as the photograph of the matchsticks may have been conducive to a more concrete reasoning, less oriented to mathematical formalisms.

### 3.3. Discussion

The statistical analyses revealed a significant difference in performance between the control and experimental groups. The presentation of the problem as a photograph of matchsticks forming the “Matchstick Arithmetic problem” was found to facilitate the process of reaching the solution. This facilitation effect, which is consistent with the misunderstanding theory, may be explained by the information conveyed by the photographic representation. In particular, the sight of two matchsticks arranged to form the plus operator more immediately conveys the possibility of decomposing not only the numbers but also the operator itself, thereby overcoming the misunderstanding that only numerical elements can be modified.

It is important to note that in this case, the informative value of the matchsticks in the photograph associated with an improvement in performance is independent of any manipulation of the matchsticks^8^. Therefore, the participant does not necessarily have to interact physically with the objects involved in a problem to achieve a better performance. More generally, even a simple representation of the physical objects, when it is able to convey the critical information needed to overcome the misunderstanding of the problem’s critical points, appears sufficient to grasp the solution.

## 4. General Conclusions

The ecological perspective on insight problem solving intuitively posits that benefits may arise from the possibility of working on these problems by acting on its physical model. Presenting a physical model of a problem would mean reasoning beyond the boundaries of the laboratory context and offering participants the opportunity to approach the task in the way they typically deal with problem situations in the real world. According to this view, the presence of objects would guide the solution process by highlighting constraints and suggesting possible actions capable of satisfying the problem’s requirements.

However, the literature also reports contradictory findings that appear to temper the evidence for the superior effectiveness of interactive formats over the traditional paper format. For example, Chuderski et al. (2021), observing no performance improvements across several insight problems, hypothesised that the benefits of physical interaction with objects primarily concern spatial insight problems. The studies presented in the present article, by contrast, show that even the manipulation of the objects themselves does not necessarily constitute an advantage, including manipulation in spatial insight problems (see the interactive condition of Experiment 1), and that the mere visual representation of objects can be beneficial to the solution process even in the absence of any physical interaction, provided that it enables the solver to overcome the misunderstanding typical of insight problems (see the picture condition of Experiment 2). Therefore, it seems appropriate to reflect critically on the actual role played by objects and by interaction with them in insight problem solving.

Attributing an *a priori* facilitating role in the solution process to physical interaction with objects on the assumption that, in everyday life, people are accustomed to solving problems through object interaction risks promoting a reductionist interpretation of the phenomenon and underestimating the human capacity to mentally represent reality. Conversely, assuming that material manipulation never offers any advantage would represent an equally simplistic explanation.

Using the misunderstanding theory as a reference framework, it may instead be more appropriate—and more sensitive to the complexity of the phenomenon—to consider the role played by objects and the possibility of manipulating them in terms of the information they are capable of conveying. Just as reformulating certain terms in a problem statement can communicate implicit ideas that allow participants to construct a representation more functional for the solution, the mere representation of real objects (the photograph of the matchsticks, in this case) can provide indirect cues useful for problem restructuring in addition to the presence of the objects themselves and their manipulation. In the “Matchstick Arithmetic problem” used in Experiment 2, the presence of overlapping matchsticks forming the plus operator helps participants understand that arithmetic symbols, as well as numbers, can be decomposed, thereby overcoming the misunderstanding that lies in the problem’s critical points.

This perspective also helps explain why, in some cases, interactive formats prove particularly effective, whereas in others they lead to performance levels comparable to those observed with the equivalent paper formats. Object manipulation would facilitate insight problem solving only when it transmits information that helps overcome the misunderstanding responsible for the formation of the problem in the solver’s mind.

In some cases, material manipulation successfully conveys information that is crucial for reaching the solution. For example, in the “Eight Coins problem” used by Chuderski et al. (2021), the participants’ attempts to move the coins can suggest the possibility of a displacement involving coin overlap, which corresponds to the solution of the problem. By contrast, in problems such as the “Pencil problem” used in Experiment 1, the presence of objects and the participants’ interactions with them are not sufficient to remove the misunderstanding. In this case, the presence of the pencils and the possibility of manipulating them does not suggest that four triangles can be formed by constructing a solid, three-dimensional figure more effectively than the paper format. As a result, participants approach the problem using the same strategy adopted in the paper condition, with no improvement in their performance.

An interesting result in this respect emerges from Experiment 1, in which STEM participants show a higher success rate in solving the problem, regardless of whether the material is manipulated. One possible explanation is that expertise in mathematics and geometry, which characterises this sample, enables participants to overcome the initial misunderstanding, on the basis of an immediate awareness of the impossibility of fulfilling the request to construct four equilateral triangles using only six edges. This awareness may facilitate (without, of course, guaranteeing) the shift towards a representation that necessarily departs from a two-dimensional one.

Finally, it is important to emphasise that the manipulation of materials undoubtedly offers the experimenter the advantage of more easily tracing the thinking processes employed by the problem solver to reach the solution, through observing their behaviour (Vallée-Tourangeau et al., 2024; Vallée-Tourangeau, 2025). This becomes even more relevant when considering the potentially deleterious effects of verbalisation on the process of solving insight problems (Schooler et al., 1993; Macchi & Bagassi, 2012; and Macchi et al., 2025). According to these authors, verbalisation tends to promote a form of problem processing that relies on conscious cognitive processes, while potentially hindering the restructuring process, which is instead at least partly unconscious and which the participants have considerable difficulty describing. For these reasons, studying the reasoning underlying the solution of an insight problem by asking participants to report it during the experiment proves problematic, whereas indirectly inferring it through the observation of solvers’ interactions with objects may represent a valid methodological alternative for investigating the restructuring process.

However, it should also be noted that adopting an ecological perspective may entail certain risks for the experimenter, particularly that of underestimating some fundamental aspects in the study of insight problem solving. Specifically, one such risk concerns neglecting the study of the problem-formation process, since the ecological perspective, by emphasising the effectiveness of interactive models, tends to focus primarily on the investigation of problem-solving strategies. While understanding the strategies adopted by participants is undoubtedly a priority in problem-solving research, it is equally important to reflect on the causes underlying the constitution of the problem itself (Mosconi, 1990; and Macchi & Bagassi, 2015). Identifying the elements responsible for the creation of the problem (problem forming) not only makes it possible to understand the critical points that must be overcome to reach the solution (problem solving) but also allows for the design of targeted experimental paradigms and applied interventions (for example, in educational contexts) aimed at facilitating comprehension and restructuring processes in the problem solver.

## Figures and Tables

**Table 1:** The percentages of correct solutions by the STEM and non-STEM participants in the standard and interactive conditions.

	Standard	Interactive	TOT
**STEM**	43.5% (N = 23)	50% (N = 22)	46.7% (N = 45)
**Non-STEM**	9.5% (N = 21)	14.3% (N = 21)	11.9% (N = 42)
**TOT**	27.3% (N = 44)	32.6% (N = 43)	

**Table 2:** Examples of the “Matchstick Arithmetic problems” and self-imposed constraints.

	Example	Solution
**Value constraint**		
The numbers cannot be decomposed.	IV = III + III	VI = III + III
**Operator constraint**		
The operators cannot be decomposed.	III = V + III	III = VI − III
**Tautology constraint**		
The operation cannot be transformed into a tautology.	III = III + III	III = III = III

## Data Availability

The data presented in this study are available at https://osf.io/q34as/overview?view_only=eecd416262ab4e79b1a7701173eb58bd (accessed on 30 April 2026).

## Notes

1 The problem asks the participant to connect four chains, each made up of three rings, with the aim of building a necklace, respecting some constraints on the number of rings that can be opened and closed.

2 These problems require the participant to correct arithmetically incorrect mathematical operations, written in Roman numerals. Both the numbers and the arithmetic signs are made up of stylised matches to correct the operation; the participant can move only one match.

3 We refer to *embodied cognition* as an umbrella label for a broader family of approaches. As Shapiro and Spaulding (2025) note, “despite the distinctions between the four “Es”—embodied, embedded, enactive, and extended—it is not uncommon to use the label “embodied” to include any or all of these “Es”. The E-fields share the view, after all, that the brain-centrism of traditional cognitive science […] stands in the way of a correct understanding of cognition” (para. 2).

4 The problem requires the participant to find a way to tie two strings hanging on opposite sides of a room, using only their arms and some available objects. The strings are long enough to be tied together, but not long enough for the participant to pick up one and reach the other on the opposite side. To solve the problem, it is necessary to tie an object to a string and use it as a weight to make it swing, so that, after having grabbed the stationary string, the participant can also grab the other during its swings.

5 The problem requires the participant to change the spatial orientation of a triangle made up of ten coins, with the constraint of being able to move only three of them.

6 The “Bacteria in the Pan problem”, the “Socks problem”, the “Flashlight and Battery problem”, the “Sheep Pen problem”, the “Matchstick Arithmetic problems”, the “Eight Coins problem”, the “Nine-Dot problem”, the “Card problem” and the “Ten-Dot problem”.

7 STEM is an acronym that refers to degree courses in which scientific–technological disciplines (science, technology, engineering, mathematics) are studied.

8 Neither the cognitive offloading hypothesis, which involves movement and surrounding physical objects to simplify information processing (Dunn & Risko, 2016; Loginov & Spiridonov, 2023), can explain our results.
